# Intrathecal Dexmedetomidine Combined With Ropivacaine in Cesarean Section: A Prospective Randomized Double-Blind Controlled Study

**DOI:** 10.3389/fmed.2022.922611

**Published:** 2022-07-07

**Authors:** Qian Zhang, Ling-yi Xia, Wei-dong Liang, Ding-yu Rao, Ping-ping Zhu, Ke-nv Huang, Zi-hao Deng, Mao-lin Zhong

**Affiliations:** ^1^First Clinical Medical College, Gannan Medical University, Ganzhou, China; ^2^Department of Anesthesiology, Nanjing Gulou Hospital Group Suqian Hospital Co., Ltd., Suqian, China; ^3^Department of Anesthesiology, The First Affiliated Hospital of Ganna Medical University, Ganzhou, China; ^4^Department of Thoracic Surgery, The First Affiliated Hospital of Ganna Medical University, Ganzhou, China

**Keywords:** cesarean section, dexmedetomidine, ropivacaine, spinal anesthesia, adverse effects

## Abstract

**Objective:**

This study aimed to find the best dose of dexmedetomidine in spinal anesthesia for cesarean section.

**Methods:**

120 American Society of Anesthesiologists (ASA) Class I and II parturients undergoing elective cesarean delivery under spinal anesthesia were randomly allocated into four groups treated with intrathecal ropivacaine (12 mg) alone (Group R) or in combination with dexmedetomidine 5 μg (Group RD1), 7.5 μg (Group RD2) and 10 μg (Group RD3). Characteristics of spinal anesthesia, hemodynamic changes, adverse effects, stress reactions and neonatal outcomes were recorded in the four groups.

**Results:**

Patients in Group RD1, RD2, and RD3 had significantly longer sustained sensory and motor block time than patients in Group R. All four groups had comparable onset times of sensory and motor block. The time for the level of sensory block to lower to S1 was longer in Group RD1 (411.07 ± 106.66 min), Group RD2 (397.03 ± 125.39 min) and Group RD3 (468.63 ± 116.43 min) than in Group R (273.60 ± 88.34 min) (*p* < 0.001). The time to recover from motor block to a Bromage score of IV was longer in Group RD1 (353.60.07 ± 137.28 min), Group RD2 (350.57 ± 118.01 min) and Group RD3 (404.67 ± 112.83 min) than in Group R (232.70 ± 93.29) (*p* < 0.01). The incidence of chills was significantly lower in the Group RD1, RD2, and RD3 than in the Group R (*p* < 0.001). There was no significant difference in the incidence of adverse effects such as hypotension, bradycardia, nausea, vomiting, hypoxemia and pruritus in the four groups (*p* > 0.05). There was no statistically significant visceral traction response or fentanyl use in the four groups (*p* > 0.05). Phenylephrine dosing was significantly higher in Group RD2 and RD3 than in Group R (*p* < 0.05), and there was no significant difference in phenylephrine dosing between Group RD1 and Group R (*p* > 0.05). There were no statistical differences in postnatal Apgar scores (1 min, 5 min after birth) (*p* > 0.05). The postoperative concentrations of β-endorphin (β-EP), cortisol (Cor) and tumor necrosis factor-α (TNF-α) in the Group RD1, RD2, and RD3 were lower than that in Group R (*p* < 0.05).

**Conclusion:**

Intrathecal 5μg of dexmedetomidine as an adjuvant to ropivacaine relieved intraoperative chills, did not increase intraoperative and postoperative adverse effects, did not increase the amount of intraoperative vasoconstrictor used, and reduced intraoperative stress reactions as well as prolonged the duration of maternal sensory and motor block, so this dose is appropriate for cesarean section.

**Clinical Trial Registration:**

[www.chictr.org.cn/], identifier [ChiCTR2200056052].

## Introduction

Spinal anesthesia is the ideal choice of anesthesia for cesarean section because of its rapid onset, safety, and effectiveness (1, 2). Spinal anesthesia can induce a strong stress reaction due to intraoperative awareness, mental tension, anxiety, and discomfort of the visceral traction response (3). Post-anesthetic chills are a common complication of spinal anesthesia (4). Therefore, to improve the quality of anesthesia, intraoperative drugs such as sedatives and analgesics are often needed to help patients reduce adverse reactions (5).

Dexmedetomidine is a highly selective α2-adrenergic agonist, and it is administered intrathecally. Dexmedetomidine acts primarily on α2 receptors at the spinal cord level. In recent years, some scholars concluded from clinical observations that intrathecal dexmedetomidine injection in a cesarean section could alleviate post-anesthetic chills, shorten the onset of lumbar anesthesia, and improve the effectiveness of local anesthetics, with no significant effects on neonates and no other adverse events (6–9). Moreover, dexmedetomidine can promote uterine contractions, so dexmedetomidine-assisted analgesia is safe after cesarean section (10).

We hypothesize that dexmedetomidine combined with ropivacaine for cesarean spinal block is safe and effective. Considering that there is less evidence to clarify the appropriate dose of intrathecal dexmedetomidine. Therefore, we designed the present prospective, randomized, double-blinded controlled study to investigate different doses of dexmedetomidine combined with ropivacaine for the cesarean spinal block. We aimed to investigate the optimal dose of dexmedetomidine in spinal anesthesia for cesarean section to provide a theoretical basis for the clinical application of dexmedetomidine.

## Methods

### Study Design

The study protocol was approved by the Research Ethics Committee of the First Affiliated Hospital of Gannan Medical College, at Ganzhou, Jiangxi Province, China (Number LLSC-2021060701) and was also registered at in a Chinese Clinical Trial Registry (ChiCTR2200056052). After arriving at the operating room, all parturients signed a written and informed consent form. All parturients were notified that dexmedetomidine had not been approved for spinal anesthesia.

One hundred and twenty parturients [singleton pregnancy; American Society of Anesthesiologists (ASA) physical status I-II; age between 20 and 45 years; gestational age ≥ 37 weeks] who were scheduled for elective cesarean section under combined spinal-epidural anesthesia from June 2021 to November 2021 were selected for the study. Parturients who had contraindications to regional anesthesia, hypertension, cardiopulmonary disease, chronic users of adrenergic receptor blockers or calcium channel blockers, and a known history of alcohol or substance abuse, multiple pregnancies, pregnancy comorbidities, allergies to any of the drugs used in the study, and a history of psychiatric disorders were excluded from the study.

Randomization was performed using a computer-generated random number code placed in a sealed envelope. Before anesthesia, an anesthesia assistant, who was not involved in subsequent anesthesia administration or data collection, prepared the anesthesia mixture based on the code number and then gave it to the anesthesiologist, who was unaware of the ratios of the drugs given. Dexmedetomidine was dissolved in saline (Dexmedetomidine Hydrochloride Injection, 2 mL:200 μg, Jiangsu Hengrui Pharmaceutical Co.) and was preservative-free and contained no additives or chemical stabilizers.

### Anesthetic Procedure

Parturients did not drink water for 6 h and fasted for 8-10 h before the operation, and they received no preoperative medication. Upon arrival at the operating room, a peripheral vein was established to allow infusion access in all parturients, and they were pre-infused 10 ml/kg of lactated Ringer’s solution, and their electrocardiogram, non-invasive blood pressure, and pulse oximetry were routinely monitored continuously. The average of the first 3 readings was considered as the basal NIBP, HR and SPO2. All parturients were administered 5 L/min of oxygen by nasal cannula. Parturients who were assessed eligible for enrollment were randomly assigned to any of the four groups. The various treatment groups were as per Table 1.

**TABLE 1:** Grouping for the study.

Group R	Intrathecal (I/T) ropivacaine 12mg (1.6 ml) + preservative free normal saline (1.4 ml)
Group RD1	I/T ropivacaine 12 mg (1.6 ml) + dexmedetomidine 5 μg (0.05 ml) + preservative free normal saline (1.35 ml)
Group RD2	I/T ropivacaine 12 mg (1.6 ml) + dexmedetomidine 7.5 μg (0.075 ml) + preservative free normal saline (1.325 ml)
Group RD3	I/T ropivacaine 12 mg (1.6 ml) + dexmedetomidine 10 μg (0.10 ml) + preservative free normal saline (1.30 ml)

*Use an insulin syringe (1 ml) to measure < 1 ml of volume.*

The mixture used for spinal anesthesia was prepared prior to anesthesia by an anesthesia assistant who was not involved in subsequent anesthesia management or data collection and was administered by a second anesthesiologist who was unaware of the mixture’s ratios and performed combined spinal-epidural anesthesia (CSEA) procedures.

During CSEA, all parturients were placed in the left lateral position, and only one puncture point, L2-3, was operated on for spinal and epidural anesthesia. After confirming the free flow of cerebrospinal fluid, the mixture was injected at the rate of 0.2ml/s (about 15 s). Then, the spinal needle was removed, and the epidural catheter was indwelled 3–4cm cephalad into the epidural space. No local anesthetic test dose was administered via the epidural catheter. No transparent cerebrospinal fluid or blood were extracted by the catheter, which confirmed that the catheter was not indwelled into the subarachnoid space and blood vessels. Then the catheter was fixed externally, and the parturient was immediately placed in the supine position with a 15-degree incline to the left to reduce the risk of supine hypotension syndrome. Data were recorded by the anesthesiologist performing the block.

After delivery, 3 ml of 2% lidocaine was added epidurally as a test dose of local anesthesia, and when there was no significant change in hemodynamics and no maternal discomfort after 5 min, the epidural catheter was connected to an epidural self-control analgesic pump with sufentanil 50 μg plus ropivacaine 150 mg which was diluted to 100 mL in saline according to the following protocol: 2.5 ml/h continuous dose, 2.5 ml/h self-controlled dose, 15 min lock time, and 10ml/h controlled maximum dose.

Intraoperative special case management: If the parturient developed a SpO2 below 90%, the parturient was given mask-assisted ventilation. If the HR was below 50 beats per minute, the parturient was given intravenous atropine 0.5 mg. If the blood pressure fell below 20% of the basal blood pressure, the parturient was given phenylephrine 40μg intravenously, and the dose could be repeated if necessary. If intraoperative visceral traction response occurred, 0.05 mg fentanyl should be injected intravenously immediately, and an additional 0.05 mg fentanyl could be added if necessary. If the parturient experienced intraoperative pain, 5 mL of 2% lidocaine was administered epidurally and repeated every 10 min if necessary, and this case was excluded from this study.

### Measurements

The onset time and duration of the sensory nerve block were recorded. Sensory nerve block onset time was defined as the time from intrathecal injection of an anesthetic solution to the absence of nociception in the T8 plane, measured every 2 min by needle prick method until the absence of nociception at the T8 level was reached. The duration of the sensory nerve block was defined as the time from the onset of the sensory block to the descent of the sensory block plane to S1, as assessed by needle pricking. After the operation, the sensory block plane was measured at 10-min intervals until the onset of decrement, then changed to 20-min intervals until the sensory block plane dropped to the S1 plane.

The onset time and duration of the motor nerve block were recorded. Motor nerve block onset time was defined as the time required from the end of intrathecal drug injection until the Bromage score reached a level I (Bromage scores: Grade I: complete block, unable to move the ankle and knee; Grade II: almost complete block, only able to move the ankle; Grade III: partial block, able to move the knee and ankle; Grade IV: no motor block). Duration of motor nerve block was defined as the time it took for the Bromage score to recover from grade I to grade IV.

When the sensory block reached the T8 plane, surgery was allowed to begin. Mean arterial pressure (MAP), heart rate (HR), and oxygen saturation (SpO2) were recorded at baseline, 5 min, 10 min, and 20 min after intrathecal administration, at delivery, and at the end of the procedure. Intraoperative adverse effects and the use of vasoactive drugs and fentanyl were recorded. The adverse effects experienced by parturients within 24 h and nerve deficits within one month were recorded after surgery. Postoperative maternal serological stress indicators were tested, such as β-endorphin (β-EP), cortisol (Cor) and tumor necrosis factor (TNF-α). The weight of the newborn and the 1 min, 5 min Apgar score were recorded. Adverse reactions such as chills, nausea and vomiting, and hypotension were also recorded. Chills grading (11): (1 = no significant muscle tension observed; 2 = mild muscle tone in the occlusal muscles; 3 = proximal muscle tremor; 4 = generalized tremor throughout the body).

### Statistical Analysis

The sample size was estimated based on the intraoperative chillis rate with a type I error of 0.05 and a power of 90%. Based on a review of the literature (2), a sample size of 23 parturients per group was necessary, and considering 15% of missing cases and dropouts, a minimum of 27 parturients per group was ultimately required. Thirty parturients were included in each group in this study. Statistical analysis of data was processed using SPSS 23.0 statistical software. Data was expressed as means and standard deviation (SD), medians and ranges, or numbers and percentages. For categorical data (ASA classification, primipara: multipara), Chi-square test or Fisher’s exact test was used. Continuous data (age, weight, height, duration of pregnancy, time of fetal delivery, duration of surgery, intraoperative blood loss, characteristics of spinal anesthesia, adverse effects, stress reactions and neonatal outcomes) were compared using analysis of variance (ANOVA). If P value was significant, then *post hoc* comparisons among the repeated measures in each group were performed by the Tukey HSD method. Differences in the hemodynamic changes were compared by repeated-measures ANOVA. The difference was considered statistically significant when *p* < 0.05.

## Results

**FIGURE 1:**
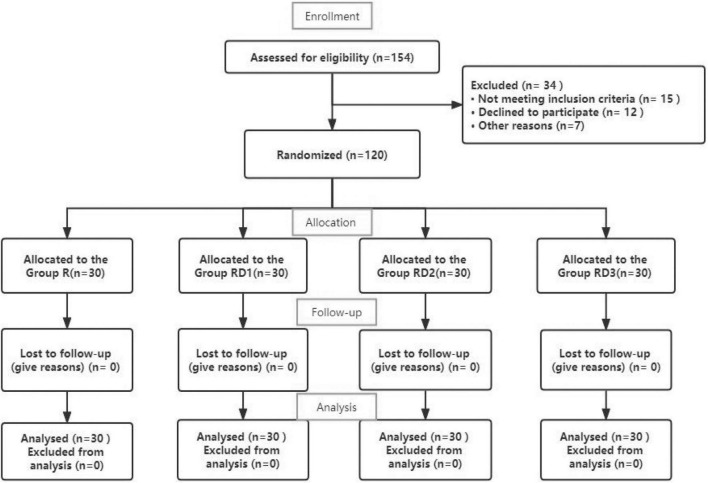
Flow diagram of study.

A total of 154 parturients entered this study. Among them, 15 cases did not meet the inclusion criteria, 12 cases did not agree to participate in the study, two cases were excluded due to epidural indwelling catheters insertion failure, intraoperative hemorrhage occurred in one case, one case had an intraoperative anaphylactic shock, and one case of anesthesia failed to reach T8 block and had to be transferred to general anesthesia. Two parturients were excluded from the trial because of additional intraoperative lidocaine remedial analgesic doses. A total of 120 patients were divided into 4 groups, and 30 patients in each group completed the experiment. The flow diagram of the study is shown in Figure 1. Parturients characteristics of the 4 groups were comparable in terms of age, weight, height and surgical characteristics. There was no significant difference in demographic, obstetric, or surgical data between the four groups (*p* > 0.05) (Table 2).

**TABLE 2:** Patients demographics and surgical characteristics.

Variable	Group R (*n* = 30)	Group RD1 (*n* = 30)	Group RD2 (*n* = 30)	Group RD3 (*n* = 30)	*P*-value
Age (years)	30.00 ± 4.43	30.00 ± 5.59	30.83 ± 4.24	28.97 ± 4.88	0.495
Height (cm)	157.23 ± 4.38	159.07 ± 3.99	157.63 ± 3.99	157.90 ± 3.84	0.338
Weight (kg)	69.30 ± 9.37	67.48 ± 6.77	67.72 ± 7.79	65.35 ± 6.34	0.262
ASA (I:II)	3:27	6:24	5:25	7:23	0.568
Pregnancy (day)	271.93 ± 2.78	272.60 ± 6.38	273.10 ± 5.00	273.60 ± 6.42	0.765
Primipara: multipara	12:18	9:21	7:23	6:24	0.328
Delivery (min)	7.57 ± 5.76	8.53 ± 3.94	9.43 ± 4.10	8.67 ± 4.34	0.759
Duration of surgery (min)	59.87 ± 19.93	55.73 ± 18.21	59.43 ± 15.83	50.70 ± 14.28	0.145
Intraoperatve blood loss(ml)	315.00 ± 60.39	293.33 ± 36.52	300.00 ± 26.26	300.00 ± 45.49	0.277

Data are presented as a number or mean ± SD. ASA = American Society of Anesthesia. Statistical significance was defined as P < 0.05. *p < 0.05 compared with Group R.

**FIGURE 2:**
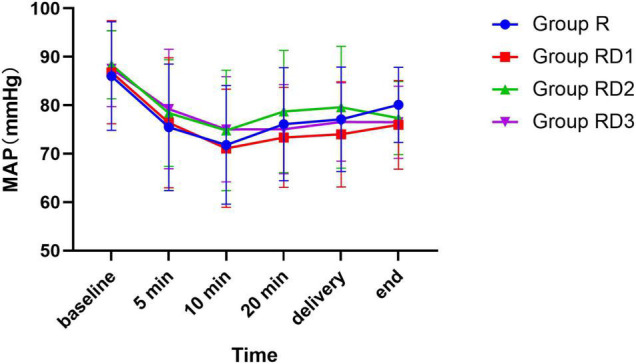
Mean arterial pressure (MAP) among the four groups. No significant differences were noted between the groups.

**FIGURE 3:**
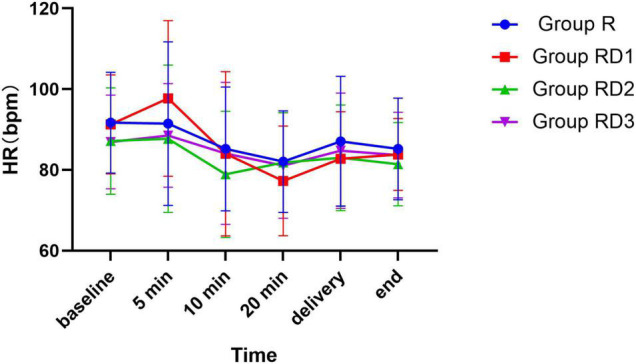
Heart rate (HR) among the four groups. No significant differences were noted between the groups.

**FIGURE 4:**
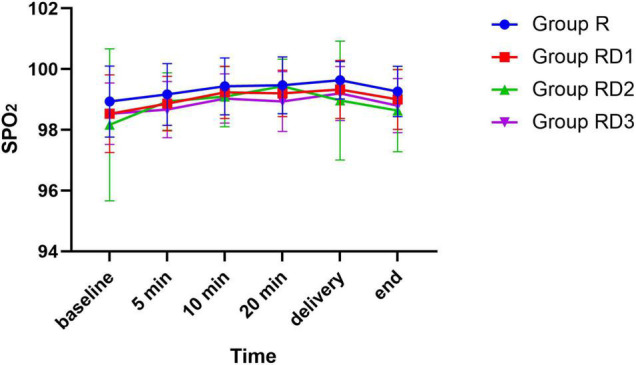
SPO_2_ among the four groups. No significant differences were noted between the groups.

The mean arterial pressure (MAP), heart rate (HR), and oxygen saturation (SPO2) were comparable across the four groups during the intraoperative period (*p* > 0.05) (Figures 2–4). No patient experienced respiratory distress at any time. Peripheral oxygen saturation was consistently greater than 95% in all patients.

**FIGURE 5:**
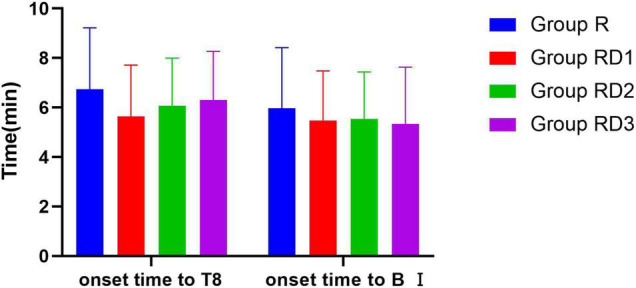
Onset time of sensory and motor block. *p < 0.05 compared with Group R.

**FIGURE 6:**
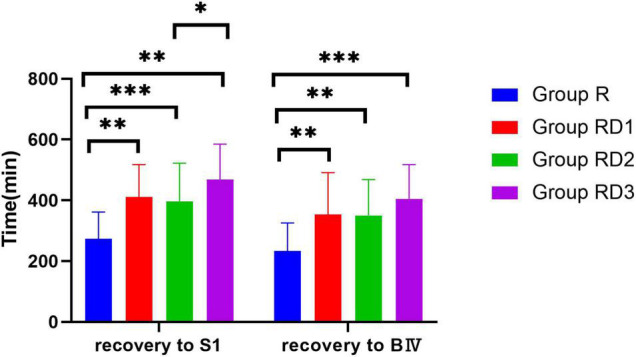
Recovery time of sensory and motor block. *p < 0.05, ^**^p < 0.01, ^***^p < 0.001.

There was no statistically significant difference in onset times of sensory and motor block in Group RD1, RD2, and RD3 compared with Group R (*p* > 0.05) (Figure 5). Patients in Group RD1, RD2, and RD3 had significantly longer sustained time of sensory and motor block than patients in Group R. The time for the level of sensory block to drop to S1 plane was longer in Group RD1 (411.07 ± 106.66 min), Group RD2 (397.03 ± 125.39 min) and Group RD3 (468.63 ± 116.43 min) than in Group R (273.60 ± 88.34 min) (*p* < 0.001). The time to recover from motor block to a Bromage score of IV was longer in Group RD1 (353.60.07 ± 137.28 min), Group RD2 (350.57 ± 118.01 min) and Group RD3 (404.67 ± 112.83 min) than in Group R (232.70 ± 93.29) (*p* < 0.01) (Figure 6).

The incidence of intraoperative chills was significantly lower in Group RD1, RD2, and RD3 than in Group R (*p* < 0.001) (Table 3). There was no significant difference in the incidence of adverse effects such as hypotension, bradycardia, nausea, vomiting, hypoxemia and pruritus in the four groups (*p* > 0.05) (Tables 3, 4). There was no statistically significant difference in visceral traction response or fentanyl use among the four groups (*p* > 0.05) (Table 3). The need for intraoperative phenylephrine dosing was significantly higher in Group RD2 and RD3 than in Group R, with P values of 0.048 and 0.010, and there was no significant difference in phenylephrine dosing between Group RD1 and Group R (*p* = 0.778) (Table 3).

**TABLE 3:** Intraoperative adverse reactions [n(%)], vasoactive drugs (times), fentanyl (mg) and neonatal conditions.

Variable	Group R (*n* = 30)	Group RD1 (*n* = 30)	Group RD2 (*n* = 30)	Group RD3 (*n* = 30)	*P*-value
Chills [n(%)]	14(46.7)	4(13.3)*	4(13.3)*	3(10.0) *	<0.001
Nausea [n(%)]	12(40.0)	9(30.0)	8(26.7)	9(30.0)	0.053
Vomiting [n(%)]	4(13.3)	5(16.7)	6(20.0)	5(16.7)	0.233
Hypotension [n(%)]	18(60.0)	17(56.7)	20(66.7)	18(60.0)	0.938
Bradycardia [n(%)]	5(16.7)	4(13.3)	9(30.0)	5(16.7)	0.373
Visceral traction response [n(%)]	7(23.3)	6(20.0)	6(20.0)	5(16.7)	0.885
Fentanyl consumption (mg)	0.50	0.35	0.35	0.3	0.797
Atropine (times)	2	2	3	3	0.935
Phenylephrine (times)	28	37	69*	73*	0.007
Newborn weight(g)	3365 ± 372	3334 ± 336	3275 ± 375	3307 ± 327	0.788
Apgar 1 min	10 (10,10)	10 (10,10)	10 (9,10)	10 (9,10)	0.295
Apgar 5 min	10 (10,10)	10 (10,10)	10 (10,10)	10 (10,10)	1

Data are presented as a number or means ± SD or median and range or numbers and percentages. Fentanyl consumption = total dose of fentanyl consumption during the operation. *p < 0.05 compared with Group R.

**TABLE 4:** Adverse events within 24 h after surgery [n(%)].

Variable	Group R (*n* = 30)	Group RD1 (*n* = 30)	Group RD2 (*n* = 30)	Group RD3 (*n* = 30)	*P*-value
Chills [n(%)]	10(33.3)	5(16.7)	4(13.3)	4(13.3)	0.196
Nausea [n(%)]	5(16.7)	6(20.0)	5(16.7)	7(23.3)	0.784
Vomiting [n(%)]	5(16.7)	4(13.3)	5(16.7)	6(20.0)	0.138
Pruritus [n(%)]	1(3.3)	0(0)	0(0)	0(0)	0.396
Hypotension [n(%)]	1(3.3)	1(3.3)	2(6.7)	0(0)	0.295
Respiratory depression [n(%)]	0(0)	0(0)	0(0)	0(0)	1
Bradycardia [n(%)]	2(6.7)	0(0)	0(0)	1(3.3)	0.547
CES [n(%)]	0(0)	0(0)	0(0)	0(0)	1
Urinary retention [n(%)]	0(0)	0(0)	0(0)	0(0)	1
PDPH [n(%)]	0(0)	0(0)	0(0)	0(0)	1
Neurological complication [n(%)]	0(0)	0(0)	0(0)	0(0)	1

Data are presented as a number. CES = Cauda Equina Syndrome. PDPH = Post dural puncture headache. *p < 0.05 compared with group R. There was no significant difference among the four groups.

None of the newborns in the four groups experienced respiratory depression, none had a heart rate below 100 beats/min, and there were no statistical differences in postnatal Apgar scores (1 min, 5 min after birth) (*p* > 0.05) (Table 3). No newborns had an Apgar score lower than 9.

The postoperative concentrations of β-endorphin (β-EP), cortisol (Cor) and tumor necrosis factor-α (TNF-α) in the Group RD1, RD2, and RD3 were lower than that in Group R (*p* < 0.001) (Table 5).

**TABLE 5:** The postoperative concentrations of β-EP,Cortisol and TNF-α.

Variable	Group R (*n* = 30)	Group RD1 (*n* = 30)	Group RD2 (*n* = 30)	Group RD3 (*n* = 30)	P-value
β-EP concentration (ng/L)	94.04 ± 4.75	90.66 ± 3.16*	88.22 ± 3.74*	84.63 ± 3.33*	<0.001
Cortisol concentration (μg/L)	273.24 ± 10.04	243.68 ± 16.66*	230.70 ± 13.45*	222.96 ± 9.62*	<0.001
TNF-α concentration (ng/L)	498.39 ± 24.91	479.30 ± 20.63*	463.30 ± 22.97*	423.57 ± 25.98*	<0.001

Data are presented as means ± SD. *p < 0.05 compared with Group R.

We followed up on the nerve deficit for another month after surgery, and no signs and symptoms of nerve deficit were found in any of the four groups.

## Discussion

In this prospective, randomized, double-blind, placebo-controlled study, we observed that the addition of 5μg, 7.5μg, or 10μg of dexmedetomidine to ropivacaine during cesarean section relieved intraoperative chills without increasing intraoperative and postoperative side effects, prolonged sensory and motor blockade, and decreased serological stress indicators. In addition, 5μg intrathecal dexmedetomidine as an adjuvant to ropivacaine did not increase the dosage of vasoactive drugs, making this dose more appropriate for cesarean delivery.

To optimize the effect of spinal anesthesia, a large number of studies have been conducted in recent years on the use of dexmedetomidine as an adjuvant to subarachnoid block in the perinatal period (12, 13). Dexmedetomidine is a highly selective, specific and potent α2-agonist (11, 14, 15). It inhibits the release of norepinephrine and blocks the transmission of pain signals to the brain center, mainly by acting on α2 receptors in the spinal cord and periphery to produce the corresponding pharmacological effects. It can also selectively stimulate the α2 adrenergic receptors in the locus coeruleus of the central nervous system, inhibit the excitability of neurons in the locus coeruleus, and thus inhibit the release of norepinephrine, and exert sedative, analgesic, anti-anxiety and anti-sympathetic effects (16, 17).

Intrathecal dexmedetomidine is a safe adjuvant, which is consistent with several studies (18, 19). Gupta et al. (18) found that intrathecal 0.5% bupivacaine 3 ml compounded with 2.5μg, 5μg or 10μg dexmedetomidine was safe and did not increase the incidence of adverse effects, and the addition of 10 μg compared with 2.5 μg or 5 μg intrathecal dexmedetomidine to 0.5% hyperbaric bupivacaine is associated with significantly earlier onset of sensory and motor block as well as prolonged duration of sensory block, motor block, analgesia, and the time difference from the offset of the motor block to the first analgesic requirement. Liu et al. (20) found that 5 μg intrathecal dexmedetomidine enhances the efficacy of spinal bupivacaine by 24% in patients undergoing cesarean section with spinal anesthesia. No additional side effect was observed by adding spinal dexmedetomidine. Tang et al. (11) concluded that 5 μg intrathecal dexmedetomidine reduced the ED50 of spinal hyperbaric ropivacaine during cesarean section by approximately 18%. In comparing the effects of intrathecal dexmedetomidine and intrathecal morphine as supplements to bupivacaine in cesarean sections under spinal anesthesia, Qi et al. (7) found that intrathecal dexmedetomidine (5 μg) significantly prolonged the motor and sensory blockade, provided a similar analgesic effect and reduced pruritus and shivering compared with morphine (100 μg). Kurhekar et al. (21) found that dexmedetomidine (2.5μg) was also effective in prolonging motor and sensory nerve blocks and reducing the incidence of pruritus with no other adverse side effects. He et al. (9) found that intrathecal dexmedetomidine (5 μg) significantly reduced the incidence and intensity of shivering induced by spinal anesthesia as an adjunct to hyperbaric bupivacaine during cesarean delivery, but intrathecal dexmedetomidine (2.5 μg) did not reduce the incidence as well as the intensity of shivering. Naaz et al. (22) showed that 10 μg of dexmedetomidine was the most optimal intrathecal dose by weighing the prolongation of anesthesia and analgesia and side effects when exploring the optimum dose of dexmedetomidine for intrathecal application in lower abdominal surgery.

We observed a significantly lower incidence of chills in parturients who used dexmedetomidine as an adjuvant to intrathecal ropivacaine than controls, consistent with previous findings (7, 9, 11). It has been suggested that dexmedetomidine reduces the occurrence of chills by suppressing the central thermoregulatory system and decreasing the perioperative stress response due to elevated adrenaline (23).

In addition, the incidence of nausea and vomiting did not differ between the groups, consistent with previous findings (5, 11). The reasons may be related to the operator’s uterine manipulation straining and peritoneal closure during cesarean section in each group or to anesthesia-induced maternal hypotension (24). Nausea and vomiting can increase the potential risk of aspiration and reduce patient satisfaction.

Interestingly, some studies suggest that dexmedetomidine may reduce the incidence of nausea and vomiting (25, 26). The mechanism might be through a reduction in sympathetic tone and catecholamine release, accelerated gastrointestinal emptying and peristalsis, and a reduction in the stimulatory effect of gastrointestinal distension on the vomiting center.

The present findings showed no statistical difference in the characteristics of spinal blocks, including the onset of sensory and motor blocks, between the four groups. These findings are contrary to those of Farokhmehr et al. (27) and similar to those of Tang et al. (11). Farokhmehr et al. (27) found that intrathecal dexmedetomidine accelerated the onset of sensory and motor block without causing side effects. All three groups with different doses of dexmedetomidine had a significant effect in prolonging the duration of the block compared to the control group. This is similar to previous findings (1, 28). The mechanism of action behind these observations has not been determined. Salgado et al. (29) suggested that the mechanism may be due to a significant synergistic effect between intrathecal dexmedetomidine and ropivacaine. Intrathecal dexmedetomidine inhibits spinal ERK1/2 signaling exhibiting potent analgesic effects in a manner dependent on α2 receptors (30). Local anesthetics work by blocking sodium channels, while dexmedetomidine works directly by acting on motor neurons or postsynaptic horn neurons in the spinothalamic pathway, thereby prolonging spinal block (31). In addition, the effects of dexmedetomidine are not limited to its interaction with a2-adrenergic receptors; its inhibitory effects on IK(DR) and INa may also affect neuronal activity *in vivo* (32). Another suggested mechanism is that dexmedetomidine acts via α2-adrenergic receptors, which subsequently induce vasoconstriction and act in this condition (33, 34).

Dexmedetomidine has been reported to have significant hemodynamic effects, causing hypotension and bradycardia as the most notable side effects (35). However, in the current study, there was no significant difference in the incidence of MAP and HR between the four groups. Dexmedetomidine did not increase the risk of bradycardia and hypotension. Yet the total amount of phenylephrine, an antihypertensive agent, was significantly higher in Group RD2 and RD3 than in Group R and RD1. It is possible that higher doses of dexmedetomidine may cause an increase in the degree of blood pressure or a prolongation of the duration of hypotension, but this is not sufficient to cause substantial adverse effects.

Hypoxemia and respiratory depression are potential side effects of dexmedetomidine. Nonetheless, no respiratory depression or hypoxemia was found in the present study, which is consistent with previous studies that intrathecal addition of dexmedetomidine 2.5μg, 5μg, and 10μg is safe and does not increase the incidence of hypoxemia and respiratory depression (18). Notably, intrathecal dexmedetomidine 15ug has been reported to possibly increase the risk associated with excessive sedation and respiratory depression (35). However, we cannot conclude that intrathecal dexmedetomidine increases the risk of hypotension, bradycardia, dyspnea, or hypoxemia. Still, adding dexmedetomidine to the maternal spinal anesthesia local anesthetics requires caution.

The present study showed that none of the neonatal Apgar scores were significantly affected by intrathecal addition of dexmedetomidine, which did not result in respiratory depression as well as heart rate slowing in neonates. This is comparable to previous studies in which intrathecal addition of dexmedetomidine at 3μg, 5μg, 7.5μg, and 10μg did not have an effect on postnatal Apgar scores (1 min, 5 min) in newborns (1, 11). The possible reason for this is that intrathecal dexmedetomidine is used in the subarachnoid space and the dose absorbed into the blood into the circulation is negligible.

Bi et al. (1) studied the effect of different doses of dexmedetomidine combined with hyperbaric ropivacaine for spinal anesthesia for cesarean delivery and found that dexmedetomidine reduced maternal postoperative c-reactive protein (CRP), interleukin-6 (IL-6) and cortisol levels, which implied a reduced maternal stress response and improved analgesia. Kang et al. (34) found that administration of dexmedetomidine during surgery reduced intraoperative and postoperative cytokine secretion as well as postoperative c-reactive protein (CRP) level and leukocyte count. Jin et al. (36) found that dexmedetomidine adjuvant to sufentanil analgesia reduced postoperative β-endorphin and cortisol levels and attenuated the postoperative stress response. Similarly, in the present study, cortisol (Cor), tumor necrosis factor (TNF)-α, and β-endorphin levels were lower in the postoperative Group RD1, RD2, and RD3 than in the postoperative Group R.

Considering the safety of dexmedetomidine for spinal anesthesia, we observed the neurological deficit one month after surgery and found no signs or symptoms of neurological deficit. The ideal adjuvant for local anesthetics would have the effect of extending the time to the endpoint without substantial risk of neurotoxicity (35). A study on the establishment of a mouse model of acute inflammation *in vivo* and *in vitro* experiments was conducted to study the neurotoxicity of dexmedetomidine on the spinal cord and cortical neurons. The results demonstrated that *in vivo* studies showed no significant pathological effects of intrathecal dexmedetomidine on the spinal cord, and *in vitro* experiments indicated a potential protective effect of dexmedetomidine against lidocaine-induced neuronal cell death (30). Human clinical studies found that intrathecal 2.5μg-10μg dexmedetomidine as an adjuvant to local anesthetics relieved patient chills and improved nerve block characteristics and did not increase the risk of adverse events in patients (1, 18).

There are some limitations of this study. First of all, it was difficult for us to really succeed in determining precisely the dose of dexmedetomidine at 5 μg (0.05 ml), 7.5 μg (0.075 ml). This should be very difficult even with 1 ml insulin syringes. This may ultimately lead to bias in the observed data for Group RD1 and RD2. Second, we did not study the effect of intrathecal adjuvant dexmedetomidine at a dose greater than 10μg on cesarean section. Third, we did not observe and record the postoperative maternal pain. Fourth, we did not record the plane at which the highest block was reached and the onset time.

## Conclusion

Under the conditions of this study, 5μg intrathecal dexmedetomidine as an adjuvant to ropivacaine to alleviate intraoperative chill response does not increase intraoperative and postoperative side effects, does not increase the dosage of vasoactive drugs, and reduced intraoperative stress reactions as well as prolongs maternal sensory and motor block, so this dosage is suitable for cesarean delivery in healthy women under spinal anesthesia.

## Data Availability Statement

All datasets generated for this study are included in the article/supplementary material.

## Ethics Statement

The studies involving human participants were reviewed and approved by the Research Ethics Committee of the First Affiliated Hospital of Gannan Medical College, at Ganzhou, Jiangxi Province, China (Number LLSC-2021060701). The patients/participants provided their written informed consent to participate in this study. Written informed consent was obtained from the individual(s) for the publication of any potentially identifiable images or data included in this article.

## Author Contributions

QZ: conceptualization, data curation, project administration, and writing – original draft. L-YX: conceptualization, data curation, and project administration. W-DL: conceptualization and project administration. D-YR: statistical analysis and writing – original draft. P-PZ, K-NH, and Z-HD: data curation and project administration. M-LZ: conceptualization, funding acquisition, data curation, project administration, and writing – review and editing. All authors contributed to the article and approved the submitted version.

## Conflict of Interest

The authors declare that the research was conducted in the absence of any commercial or financial relationships that could be construed as a potential conflict of interest.

## Publisher’s Note

All claims expressed in this article are solely those of the authors and do not necessarily represent those of their affiliated organizations, or those of the publisher, the editors and the reviewers. Any product that may be evaluated in this article, or claim that may be made by its manufacturer, is not guaranteed or endorsed by the publisher.

## References

[1] Bi YH Wu JM Zhang YZ Zhang RQ. Effect of different doses of intrathecal dexmedetomidine as an adjuvant combined with hyperbaric ropivacaine in patients undergoing cesarean section. *Front Pharmacol.* (2020) 11:342. 10.3389/fphar.2020.00342 32265713 PMC7098998

[2] Hirose N Kondo Y Maeda T Matsui M Matsuda M Suzuki T. Prophylactic infusion of phenylephrine is effective in attenuating the decrease in regional cerebral blood volume and oxygenation during spinal anesthesia for cesarean section. *Int J Obstet Anesth.* (2019) 37:36–44. 10.1016/j.ijoa.2018.09.006 30482720

[3] Han FP. Feasibility study of dexmedetomidine for sedation in spinal canal anesthesia for lower uterine cesarean delivery. *China Pract Med.* (2018) 13:150–1. 10.14163/j.cnki.11-5547/r.2018.33.087

[4] Zhao JS. Dose-effect relationship of single loading dexmedetomidine in sedation for intravertebral anesthesia in patients undergoing cesarean delivery. *Jiangxi Med J.* (2019) 54:77. 10.3969/j.issn.1006-2238.2019.12.061

[5] Yang MJ Wang LY Chen H Chen XZ. (2018). Efficacy of dexmedetomidine as a neuraxial adjuvant for elective cesarean sections: a meta-analysis of randomized trials. *Int J Clin Exp.* (2018) 11:8855–64.

[6] Shen QH Li HF Zhou XY Yuan XZ Lu YP. Dexmedetomidine as an adjuvant for single spinal anesthesia in patients undergoing cesarean section: a system review and meta-analysis. *J Int Med Res.* (2020) 48:300060520913423. 10.1177/0300060520913423 32466699 PMC7263150

[7] Qi XF Chen DL Li GH Huang XL Li YT Wang XG Comparison of intrathecal dexmedetomidine with morphine as adjuvants in cesarean sections. *Biol Pharm Bull.* (2016) 39:1455–60. 10.1248/bpb.b16-00145 27349272

[8] Crespo S Dangelser G Haller G. Intrathecal clonidine as an adjuvant for neuraxial anaesthesia during caesarean delivery: a systematic review and meta-analysis of randomised trials. *Int J Obstet Anesth.* (2017) 32:64–76. 10.1016/j.ijoa.2017.06.009 28823524

[9] He L Xu JM Liu SM Chen ZJ Li X Zhu R. Intrathecal dexmedetomidine alleviates shivering during cesarean delivery under spinal anesthesia. *Biol Pharm Bull.* (2017) 40:169–73. 10.1248/bpb.b16-00651 28154256

[10] Wu XM Xue ZZ Ma H Wang G Shi XY Huang SQ Expert consensus on the clinical application of dexmedetomidine (2018). *J Clin Anesthesiol.* (2018) 34:820–3. 10.12089/jca.2018.08.024

[11] Tang YW Yang MJ Fu F Huang XD Feng Y Chen XZ. Comparison of the ED50 of intrathecal hyperbaric ropivacaine co-administered with or without intrathecal dexmedetomidine for cesarean section: a prospective, double-blinded, randomized dose-response trial using up-down sequential allocation method. *J Clin Anesth.* (2020) 62:109725. 10.1016/j.jclinane.2020.109725 32036258

[12] Vatsalya T Waikar C Singh M. Comparison of intravenous bolus and infusion of dexmedetomidine on characteristics of subarachnoid block. *Anesth Essays Res.* (2018) 12:190–3. 10.4103/aer.AER_111_17 29628580 PMC5872862

[13] Dolma L Salhotra R Rautela RS Banerjee A. Isobaric ropivacaine with or without dexmedetomidine for surgery of neck femur fracture under subarachnoid block. *J Anaesthesiol Clin Pharmacol.* (2018) 34:518–23. 10.4103/joacp.JOACP_226_18 30774234 PMC6360896

[14] Grape S Kirkham KR Frauenknecht J Albrecht E. Intra-operative analgesia with remifentanil vs. dexmedetomidine: a systematic review and meta-analysis with trial sequential analysis. *Anaesthesia.* (2019) 74:793–800. 10.1111/anae.14657 30950522

[15] Zhang SH Zheng GZ Li XY. Dexmedetomidine combined with clonoxicam in post-laparoscopic hysterectomy analgesia. *China J Mod Med.* (2018) 28:122–6. 10.3969/j.issn.1005-8982.2018.13.023

[16] Shime N. Early extubation for infant cardiac surgery supported by dexmedetomidine use. *Pediatr Crit Care Med.* (2019) 20:989–90. 10.1097/PCC.0000000000002031 31580274

[17] Wang QM Wei LM. Dexmedetomidine compounded with sufentanil for postoperative analgesia in gynecological laparoscopy. *Jiangxi Med J.* (2019) 54:668–70. 10.3969/j.issn.1006-2238.2019.6.031

[18] Gupta M Gupta P Singh DK. Effect of 3 different doses of intrathecal dexmedetomidine (2.5 μg, 5μg, and 10 μg) on subarachnoid block characteristics: a prospective randomized double blind dose-response trial. *Pain Phys.* (2016) 19:E411–20. 27008297

[19] Mohamed SA El-Rahman AM Fares KM. Intrathecal dexmedetomidine, ketamine, and their combination added to bupivacaine for postoperative analgesia in major abdominal cancer surgery. *Pain Phys.* (2016) 19:E829–39. 27454273

[20] Liu L Qian J Shen B Xiao F Shen HX. Intrathecal dexmedetomidine can decrease the 95% effective dose of bupivacaine in spinal anesthesia for cesarean section: a prospective, double-blinded, randomized study. *Medicine (Baltimore).* (2019) 98:e14666. 10.1097/MD.0000000000014666 30817591 PMC6831281

[21] Kurhekar P Kumar SM Sampath D. Comparative evaluation of intrathecal morphine and intrathecal dexmedetomidine in patients undergoing gynaecological surgeries under spinal anaesthesia: a prospective randomised double blind study. *Indian J Anaesth.* (2016) 60:382–7. 10.4103/0019-5049.183387 27330198 PMC4910476

[22] Naaz S Bandey J Ozair E Asghar A. Optimal dose of intrathecal dexmedetomidine in lower abdominal surgeries in average indian adult. *J Clin Diagn Res.* (2016) 10:UC09–13. 10.7860/JCDR/2016/18008.7611 27190922 PMC4866220

[23] Fang AL Li Y Lu GJ Ma J. The analgesic effect and adverse reactions of dexmedetomidine in women undergoing elective cesarean section using spinal or spinal-epidural anesthesia. *China Med.* (2020) 15:1088–92. 10.3760/j.issn.1673-4777.2020.07.028 30704229

[24] Bai X Yang Y Xia CL. Interpretation of guidelines for postoperative care in cesarean delivery: enhanced recovery after surgery(ERAS)society recommendations. *Chine Nurs Res.* (2020) 34:1493–6. 10.12102/j.issn.1009-6493.2020.09.001

[25] Hu BL Zhou HY Zou XH Shi J Li XY Tan LA. Comparison of dexmedetomidine and midazolam for the prevention of postoperative nausea and vomiting caused by hemabate in cesarean delivery: a randomized controlled trial. *Drug Des Devel Ther.* (2020) 14:2127–33. 10.2147/DDDT.S251525 32546975 PMC7266306

[26] Liu Y Chen HX Kang DL Kuang XH Liu WX Ni J. Influence of dexmedetomidine on incidence of adverse reactions introduced by hemabate in postpartum hemorrhage during cesarean section. *Int J Clin Exp Med.* (2015) 8:13776–82. 26550325 PMC4613010

[27] Farokhmehr L Modir H Yazdi B Kamali A Almasi-Hashiani A. Effect of different doses of intrathecal dexmedetomidine on hemodynamic parameters and block characteristics after ropivacaine spinal anesthesia in lower-limb orthopedic surgery: a randomized clinical trial. *Med Gas Res.* (2019) 9:55–61. 10.4103/2045-9912.260645 31249252 PMC6607861

[28] Safari F Aminnejad R Mohajerani SA Farivar F Mottaghi K Safdari H. Intrathecal dexmedetomidine and fentanyl as adjuvant to bupivacaine on duration of spinal block in addicted patients. *Anesth Pain Med.* (2016) 6:e26714. 10.5812/aapm.26714 27110524 PMC4837787

[29] Salgado PF Sabbag AT Silva PC Brienze SL Dalto HP Módolo NS Efeito sinérgico entre a dexmedetomidina e a ropivacaína 0,75% na anestesia peridural [Synergistic effect between dexmedetomidine and 0.75% ropivacaine in epidural anesthesia]. *Rev Assoc Med Bras.* (2008) 54:110–5. 10.1590/s0104-42302008000200011 18506317

[30] Zhang HX Zhou F Li C Kong M Liu H Zhang P Molecular mechanisms underlying the analgesic property of intrathecal dexmedetomidine and its neurotoxicity evaluation: an in vivo and in vitro experimental study. *PLoS One.* (2013) 8:e55556. 10.1371/journal.pone.0055556 23409000 PMC3567091

[31] Mahendru V Tewari A Katyal S Grewal A Singh MR Katyal R. A comparison of intrathecal dexmedetomidine, clonidine, and fentanyl as adjuvants to hyperbaric bupivacaine for lower limb surgery: a double blind controlled study. *J Anaesthesiol Clin Pharmacol.* (2013) 29:496–502. 10.4103/0970-9185.119151 24249987 PMC3819844

[32] Chen BS Peng H Wu SN. Dexmedetomidine, an alpha2-adrenergic agonist, inhibits neuronal delayed-rectifier potassium current and sodium current. *Br J Anaesth.* (2009) 103:244–54. 10.1093/bja/aep107 19542547

[33] El-Hennawy AM Abd-Elwahab AM Abd-Elmaksoud AM El-Ozairy HS Boulis SR. Addition of clonidine or dexmedetomidine to bupivacaine prolongs caudal analgesia in children. *Br J Anaesth.* (2009) 103:268–74. 10.1093/bja/aep159 19541679

[34] Kang SH Kim YS Hong TH Chae MS Cho ML Her YM Effects of dexmedetomidine on inflammatory responses in patients undergoing laparoscopic cholecystectomy. *Acta Anaesthesiol Scand.* (2013) 57:480–7. 10.1111/aas.12039 23240685

[35] Eid HE Shafie MA Youssef H. Dose-related prolongation of hyperbaric bupivacaine spinal anesthesia by dexmedetomidine. *Ain Shams J Anesthesiol.* (2011) 4:83–95. 10.7860/JCDR/2017/26241.9654 28571237 PMC5449883

[36] Jin D Bai Y Wu H. Effects of dexmedetomidine used to supplement analgesia with sufentanil on stress response and inflammatory response after cardiac valve replacement with CPB. *Chinese J Anesthesiol.* (2016) 36:49–52. 10.3760/cma.j.issn.0254-1416.2016.01.014 30704229

